# TNFR1 signaling is positively regulated by Jak-2 and c-Src via tyrosine phosphorylation

**DOI:** 10.55730/1300-0152.2677

**Published:** 2023-11-06

**Authors:** Fatma Zehra HAPİL ZEVKLİLER, Fatma Ece ÇOPUROĞLU, Mustafa Gökhan ERTOSUN, Ufuk MERT, Derya ÖZEŞ, Osman Nidai ÖZEŞ

**Affiliations:** 1 Department of Medical Biology and Genetics, Akdeniz University, Antalya, Turkiye; 2 Department of Reconstructive Surgery, Akdeniz University, Antalya, Turkiye; 3 Atatürk Health Care Vocational School, Ege University, İzmir, Turkiye; 4 ALTAY Biopharma, San Bruno, CA, USA

**Keywords:** TNF, TNFR1, JAK2, c-Src, ERK, Akt

## Abstract

**Background/aim:**

Tumor necrosis factor alpha (TNFα, a.k.a. TNF) is a pleiotropic cytokine that exerts most of its effects through type 1 TNF receptor (TNFR1). Following TNF binding, TNFR1 recruits TRADD (tumor necrosis factor receptor type 1-associated DEATH domain). This interaction triggers formation of signalosome complexes which have been claimed to induce apoptosis (via downstream caspase activations), inflammation (via NF-kappaB) and stress pathways (JNK & p38). However, the mechanism underlying TNF-induced ERK and AKT activation is not completely revealed. TNFR1 is known to constitutively bind c-Src and JAK2, and these enzymes were previously demonstrated to modulate TNF signaling. Therefore, we hypothesized that TNFR1 could be tyrosine phosphorylated by JAK2 and/or c-Src and TNF-induced ERK and Akt activation may be mediated by this phosphorylation.

**Materials and methods:**

Site-directed mutagenesis (SDM) was performed to substitute the two putative Tyrosine phosphorylation sites on TNFR1 (Y360 and Y401) with alanine (A) or with aspartic acid (D), to inhibit or mimic constitutive phosphorylation, respectively. In 293T cells transfected with mutated or wild type TNFR1, ERK and Akt activations were determined by western blot. TNFR1 interaction with c-Src, JAK2, p85 and Grb2 was examined by co-IP. NF-kB activation was measured by luciferase assay, while proliferation was measured by MTT and apoptosis was evaluated by colorimetric caspase 8/3 assays. For determination of necrosis rates, cellular DNA fragmentation ELISA was performed.

**Results:**

In this report, we show that TNFR1 is phosphorylated by JAK2 tyrosine kinase at Y401 and by c-Src at Y360 and Y401. Phosphorylation of Y360 and Y401 augments the interaction of Grb2 and PI3Kp85 with TNFR1. We also demonstrate that phosphomimetic mutations of Y360D and Y401D enhance ERK and Akt activation.

**Conclusion:**

TNFR1 is tyrosine phosphorylated by both c-Src and JAK2, triggering a “noncanonical” pathway, that activates ERK and Akt.

## 1. Introduction

Tumor necrosis factor alpha (TNF) is a pleiotropic cytokine with significant roles in activation of immunity and inflammation, as well as in growth and differentiation of both transformed and nontransformed cells (Balkwill et al., 2005; Hapil et al., 2020). TNF exerts its biological effects by binding to two different receptors, TNFR1 and TNFR2, of which the former is ubiquitously expressed. The expression of the latter is known to be particularly cell type-dependent (Yang et al., 2019). TNFR1 is a 55 kD transmembrane protein with a death domain (DD) in its cytoplasmic region (aa 356–441) (Tartaglia et al., 1993) and this domain, by way of protein-protein interactions, is responsible for most effects of TNF. The proteins that are shown to interact with the DD of TNFR1 are TNF-receptor associated death domain (TRADD) (Hsu et al., 1995; Hsu et al., 1996) and receptor-interacting protein-1 (RIP1)(Hsu et al., 1996; Ting et al., 1996), which bind via death domain-death domain (DD-DD) interactions. TRADD binding to TNFR1 recruits the Fas-associated death domain (FADD) and procaspase 8 into a complex that initiates apoptotic caspase cascade, while binding of TRADD to RIP1 and TNFR-associated factor 2 (TRAF2) induces the formation of complex-1 (Zheng et al., 2006). Complex-1 is composed of RIP1, TNF-receptor associated factor-2 (TRAF2), cellular inhibitor of apoptosis protein 1/2 (cIAP1/2), linear ubiquitin chain assembly complex (LUBAC), and transforming growth factor-activated kinase-1 (TAK1) (Hsu et al., 1996; Jackson Bernitsas et al., 2007). Stabilization of this complex and amplification of downstream signals are ensured by Met1-linked linear ubiquitin chains that are added by LUBAC. TAK1 initiates activation cascade of p38 and JNK mitogen-activated protein kinases (MAPK), whereas activation of IKK initiates the canonical NF-kB pathway (Hsu et al., 1996; Kanayama et al., 2004; Bertrand et al., 2008).

As summarized above, most of our knowledge on TNFR1 signaling depends on DD-DD interactions. On the other hand, receptors for the other cytokines, such as IL-6, which resembles to TNF in its pleiotropic nature (Kishimoto 2006), exert signals via binding of adaptor proteins to tyrosine phosphorylated residues. We have previously shown the interaction of TNFR1 with c-Src and JAK2 (Pincheira et al., 2008). Here, we questioned whether TNFR1 exerted a signaling pathway starting with tyrosine phosphorylation of the receptor, and we showed that JAK2 and c-SRC directly phosphorylated TNFR1 and these phosphorylation events potentiated TNF-induced activations of ERKs and Akt kinases.

## 2. Materials and methods

### 2.1 Antibodies and reagents

We purchased monoclonal primary antibodies for TNFR1 (sc-8436), JAK2 (sc-294), pERK (sc-7383), ERK (sc-94), pY20 (sc-508), Grb2(sc-255), c-Src (sc-8056), GAPDH (sc-47724), His-probe (sc-803) from Santa Cruz Biotechnology, and pAkt (9271, 9275), Akt (9272) and p85 (13666S) from Cell Signaling Technology. HRP-conjugated secondary antibodies against mouse and rabbit IgG were KPL (474–1806 and 474–1506, respectively). Standard mouse and rabbit IgG that we used in immunoprecipitation experiments were purchased from Santa Cruz Biotechnology, recombinant human TNF was purchased from Sigma (H8916), JAK2-inhibitor AZD1480 (sc364735) and protein A/G-Agarose beads (sc-2003) were purchased from Santa Cruz, and c-Src inhibitor PP1 was purchased from BioMol (cay-14244). Western Blot detection substrate Clarity ECL was from Bio-Rad (1705061).

### 2.2 Plasmids and mutagenesis

TNFR1 plasmid was previously created by cloning into pcDNA3.1A backbone (Hapil et al., 2020). Site-directed mutagenesis was performed by Pfu polymerase (Agilent Stratagene) reaction using the primers listed in Table 1. PCR products were digested with DpnI digestion enzyme (NEB) and transformed into competent *E.coli*. Next day, plasmid miniprep was performed with phenol-chloroform extraction method and plasmid sequences were verified by Sanger sequencing using Applied Biosystems 3130 XL.

### 2.3. Cell culture, transfections and treatments

293T cells (passage 15) were cultured under standard cell culture conditions in high glucose DMEM with 10% Fetal Bovine Serum (GIBCO) and 1% Penicillin/streptomycin/amphotericin B (Biological Industries). For most of the experiments, transfections were made using calcium phosphate precipitation, while in functional tests Lipofectamine 2000 (Thermo Scientific) reverse transfection was performed as described by the manufacturer. TNFR1 overexpression after transfections was validated by Western Blot. Prior to all treatments with TNF or inhibitors, cells were serum-starved in serum free media for 16 h and then treated with 100 μM Sodium orthovanadate (Na_3_VO_4_) for 1 h. Inhibitor doses were as follows: 0.26 nM AZD1480 for 30 min or 200 nM PP1 for 30 min prior to TNF treatments. TNF treatment duration varied among experiments, therefore is indicated in the results section.

### 2.4. Immunoprecipitation and Western Blot

Immunoprecipitation experiments were performed with protein A/G-Agarose beads, with 2μg primary antibody per mg of protein lysates, which were determined by Bradford method. Both immunoprecipitation eluates and cellular lysates were run on SDS-PAGE, wet-transferred to Immobilion-P PVDF membranes and blotted as described before (Hapil et al., 2020)

### 2.5. In vitro kinase reactions

JAK2 and c-Src were immunoprecipitated from untransfected 293T cells, either treated with 10ng/mL TNF or left untreated for 30 min; while TNFR1 was immunoprecipitated from TNFR1 transfected 293T cells that were not treated with TNF. Reaction mixture for JAK2 was 5 mM MnCl_2_, 5 mM MgCl_2_, 60 mM HEPES, 3 μM Na_3_VO_4_, 1.25 mM DTT, and the reaction mixture for c-Src was 5 mM MOPS, 2.5 mM β-glycerolphosphate, 1mM EGTA, 0.4 mM EDTA, 4 mM MgCl_2_, 2.5 mM MnCl_2_, 0.5 mM DTT, 2.5 μM Na_3_VO_4_. TNFR1 and enzymes were mixed and washed in reaction mixtures for at least three times by centrifugation at 6000 xg for 1 min. 10 μM ATP was used in 50 μL reaction, the concentration of AZD1480 was 0.26 nM and PP1 was 200 nM in the inhibition tube.

### 2.6. Proliferation and apoptosis tests

For caspase activation tests, cellular lysates were obtained from 24 h TNF treated cells. Then, colorimetric Caspase-3 and Caspase-8 activation tests (Biovision K106-200; Biovision K113-200) were performed as described by the manufacturer. Necrosis and/or late-apoptosis rates were analyzed with Cellular DNA Fragmentation ELISA by measuring fragmented DNA released to supernatant (Merck-Roche, 11585045001).

For measurement of proliferation, cells were reverse-transfected using Lipofectamine-2000 (Thermo Scientific) in 96-well plates, with at least 6 repeats for each vector. After 24 h of culture, media were replaced either with final 10 ng/mL TNF concentration or without TNF. Following another 24 h of incubation, 20 μL MTT reagent was added into wells and left for 6 h of incubation. Then MTT precipitates was dissolved in 100 μL DMSO and colorimetric measurements were performed in 540 and 690 nm wavelengths, using Multiskan Spectrum (Thermo Scientific).

### 2.7. Determination of NF-κB activation

150 ng NF-luc plasmid and 150 ng of either wild-type or mutant TNFR1 plasmids were mixed and 293T cells were reverse transfected in 96-well plates. Twenty-four h after transfection, wells were supplemented with either fresh media or fresh media containing TNF with the final concentration 10 ng/mL. Following 6 h of incubation, luciferase activity was measured by using OneGlo Luciferase Assay (Promega), for 20 ms in Fluoroskan Ascent Luminometer. Luciferase activity scores were normalized with simultaneously performed MTT readings.

### 2.8. Statistical analysis

ImageJ software was utilized for measuring Western Blot band intensities. For determination of protein phosphorylation levels, protein phospho-form band intensities were proportioned to total protein band intensities. Graphs were expressed as Means±SEM and statistical analysis was performed by GraphPad Prism Software.

## 3. Results

### 3.1. TNFR1 is phosphorylated by JAK2 and c-Src

**Figure 1:**
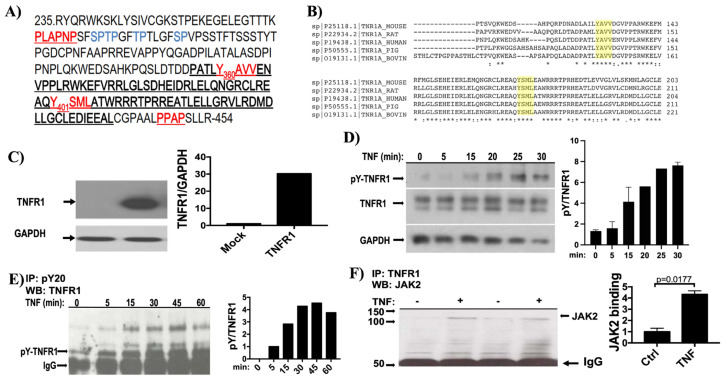
TNFR1 is tyrosine phosphorylated. a) aa sequence of TNFR1 cytoplasmic domain. Underlined bold sequence is the death domain (aa 356–441). Within the death domain, putative tyrosine (Y360, Y401) sites are marked in red. Between aa 266–271, JAK2 binding consensus sequence (PxxPxP) and between aa 448–451, SH3 binding consensus sequence (PxxP) are marked in red. b) Putative tyrosine phosphorylation sites Y360 and Y401 were conserved among species. Amino acid sequence of TNFR1 cytoplasmic domain of human, mouse, rat, bovine and pig were aligned by Clustal Omega (Sievers et al., 2011) server. Putative phosphorylation sites that are highlighted where conserved. c) 293T cells were transfected with Mock or with TNFR1 expression vector followed by incubation for 24 h, and level of TNFR1 was determined by western blot analysis, and fold change in expression was determined by dividing band intensity of TNFR1 by that of GAPDH. d) TNFR1-transfected 293T cells were treated with TNF for indicated time points. Cellular lysates were analyzed with phospho-tyrosine (pY20) western blot (upper panel), blot was stripped off and labeled with TNFR1 (medium panel) and GAPDH (lower panel). To analyze data, phospho-tyrosine band intensities were divided by TNFR1 band intensities, and graph was generated by GraphPad Prism software. e) Tyrosine phosphorylated proteins were immunoprecipitated from TNFR1 transfected cells treated with TNF for indicated time points. Immunoprecipitation samples were probed with TNFR1 western blot. Arrow indicates expected TNFR1 band according to molecular weight marker. For graphical analysis, TNFR1 band intensities were proportioned to IgG. f) To explore the physical interaction between TNFR1 and JAK2, TNFR1 was immunoprecipitated from 1 mg total lysates obtained in the presence or absence of TNF and blotted for JAK2. To ensure equal TNFR1 expression, transfected cells were divided into four plates, 2 of which were treated with 10ng/mL TNF for 15 min and remaining 2 left untreated. For immunoprecipitation, we used equal amounts of protein (1 mg) for all 4 samples. At the bottom of the blot, arrow-marked IgG bands validate equal loading.

TNFR1 possesses the (P_266_LAPNP_271_) sequence, (Figure 1a) which contains the consensus JAK binding site (PxxPxP) (Tanner et al., 1995). In addition to this, when we inspected human TNFR1 amino acid sequence (NCBI accession number AAA61201.1), we identified three putative tyrosine phosphorylation sites: Y318, Y360, and Y401. Among these, Y360 and Y401 were in the death domain of TNFR1 and conserved among species (Figure 1b). Importantly, Y360 and Y401 were reported to be phosphorylated according to the PhosphositePlus database (Hornbeck et al., 2015). In order to confirm these phosphorylations, TNFR1 was ectopically expressed nearly 25–30 fold (Figure 1c) relative to endogenous TNFR1 in 293T for 24 h. Then the cells were treated with TNF (10 ng/ml) for 0, 5, 15, 20, 25 and 30 minutes in the presence of phosphatase inhibitor Na_3_VO_4_ (100 uM). Tyrosine phosphorylation of TNFR1 was detected within 15 min of post TNF treatment, and increased until 30 min (Figure 1d). The level of phosphorylation observed in 30 min post-TNF treatment is retained over time (data not shown). These results were validated by immunoprecipitating TNFR1 with antiphosphotyrosine antibody (PY20) and blotting with anti-TNFR1 antibody (Figure 1e). Importantly, a significant increase in JAK2 binding to TNFR1 was also observed in TNF-stimulated cells (Figure 1f).

**Figure 2:**
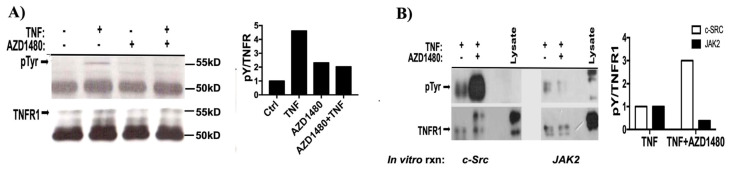
TNFR1 is directly phosphorylated by JAK2 and c-Src. a) JAK2 in vitro kinase reaction was performed with JAK2 immunoprecipitated from TNF treated or untreated 293T cells and TNFR1 immunoprecipitated from TNFR1 transfected 293T cells without TNF treatment. AZD1480 was added into separate reaction tubes to inhibit JAK2 activity. b) c-Src (left) and JAK2 (right) in vitro kinase reactions in the presence of AZD1480. Phospho-tyrosine immunoblotting was performed with pY-HRP antibody to eliminate IgG bands. Graphs represent two independent experiments.

Since we have previously shown that c-Src and JAK2 interact with TNFR1(Pincheira et al., 2008), we postulated that Y360 and Y401 could be a target for JAK2 and c-Src-mediated phosphorylation. To demonstrate direct phosphorylation of TNFR1 by JAK2, we performed an in vitro kinase assay with JAK2 immunoprecipitated from 293T cells that are treated or not treated with TNF (10 ng/mL). We performed an in vitro kinase reaction in the presence or absence of JAK2 inhibitor AZD1480. TNFR1 was immunoprecipitated from serum-starved 293 T cells ectopically expressing wild type TNFR1. As shown in Figure 2a, JAK2 effectively phosphorylated wild type TNFR1 and this was reduced by half in the presence of AZD1480. Since TNFR1 was previously demonstrated to bind both JAK2 and c-Src, we sought to determine whether JAK2 activation preceded c-Src-mediated phosphorylation. To identify the sequential order of phosphorylation events, c-Src and JAK2 were immunoprecipitated from 293T cells treated with TNF or AZD1480 and an in vitro kinase reaction was performed using the TNFR1 as substrate. Treatment with AZD1480 decreased JAK2-mediated TNFR1 phosphorylation by nearly 50% whereas it increased c-Src-mediated phosphorylation (Figure 2b). These results indicate that TNFR1 is phosphorylated by both JAK2 and c-Src, and JAK2-mediated phosphorylation has an inhibitory effect on c-SRC-mediated TNFR1 phosphorylation.

### 3.2. TNFR1 tyrosine phosphorylation on Y401 residue potentiates ERK activation

As we demonstrated tyrosine phosphorylation of TNFR1 by both JAK2 and c-Src, we next questioned whether these phosphorylation events play role in activations of ERK or Akt pathways. Growth factor receptors activate Grb2-Ras-Raf-ERK pathway and activated Ras can also activate PI3K pathway by binding to PI3Kp110 (Rodriguez Viciana et al., 1996). In addition to growth factor receptors, TNF also activates ERK pathway by recruiting Grb2 to PPAP motif at the carboxy terminal of TNFR1 (Copuroglu et al., 2021). Although Grb2 interaction with TNFR1 was prerequisite for the formation of TNFR1/Grb2/Ras signaling, it was not sufficient for full activation of c-Raf-1 kinase (Tartaglia et al., 1993). This led us to postulate that tyrosine phosphorylation sites of TNFR1 could be used as docking sites for Grb2 binding and subsequent activation of Grb2-Ras-ERK signaling pathway. Therefore, we generated site-mutants of TNFR1 to mimic and inhibit phosphorylation at putative tyrosine phosphorylation sites (Y360 and Y401) on the death domain, as listed in Table 2.

**Figure 3:**
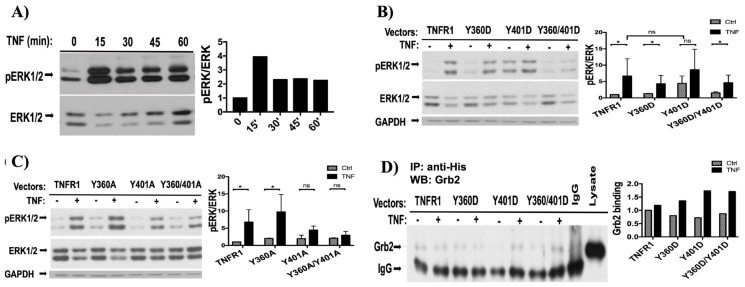
TNFR1 mediated ERK activation is mediated through Y401 phosphorylation. a) Pattern of TNF-induced ERK activation was explored by western blot of lysates obtained from 293T cells treated with TNF for the indicated time points. b) pERK and ERK immunoblotting was performed on lysates obtained from 293T cells transfected with aspartic acid mutants of tyrosine phosphorylation sites. c) pERK and ERK immunoblotting was performed on lysates obtained from 293T cells transfected with Alanine mutants of tyrosine phosphorylation sites. d) Wild type and phosphomimetic mutants of TNFR1 ectopically expressed in 293T cells treated with or without 10ng/mL TNF were immunoprecipitated and blots were labeled with anti-Grb2 antibody. To determine fold change in binding of Grb2, band intensities of Grb2 was divided by that of IgG**_25_**. For a, b, and c ERK activation was determined by dividing pERK band intensities by ERK band intensities. _*_: p < 0.05, _**_: p < 0.01, ns: p > 0.05.

At first, we determined the time-course of TNFα-induced ERK activation. As shown in Figure 3a, and also in our previous study (Hapil et al., 2020), TNFα induced 4-fold ERK activation within 15 min, therefore, the influence of our mutants on ERK phosphorylation were tested after 15 min of TNF stimulation. As shown in Figure 3b, Y360D mutant slightly increased background ERK phosphorylation, however ectopic expression of Y401D mutant, without a need for TNF treatment, increased ERK phosphorylation level similar to TNFα-induced ERK phosphorylation levels. Also, ERK pathway activation in Y401D-expressing cells were not TNF-responsive. ERK activation levels of Y360D/Y401D double mutant was parallel with the levels observed in Y360D mutant-transfected cells, which indicates a suppressive effect of Y360D on ERK activation. These results strongly suggest that Y401 phosphorylation play a significant role in activation of Ras-ERK pathway and Y360 phosphorylation seem to interfere with this.

To give further support for our findings, we determined ERK activation in 293T cells ectopically expressing Alanine mutants of TNFR1. As shown in Figure 3c, ectopic expression of Y360A significantly potentiated TNF-induced ERK phosphorylation whereas ERK phosphorylation was robustly diminished in Y401A and Y360A/401A expressing cells. These results show that in cells expressing Y401D mutant, ERK activation was elevated even in the absence of TNF treatment (Figure 3b) while in Y401A mutant expressing cells, TNF-dependent ERK activation was suppressed (Figure 3c). Therefore, the phosphorylation of TNFR1 at Y401 seems to promote TNFα-mediated ERK phosphorylation. On the other hand, TNF-induced ERK activation was suppressed in cells expressing Y360D mutant (Figure 3b), and was further potentiated in cells expressing Y360A mutant (Figure 3c), indicating a suppressive role for TNFR1 Y360 phosphorylation on TNF-induced ERK activation. To further confirm our findings and published results (Tartaglia et al., 1993), we explored the influence of Y360D and Y401D mutants on Grb2-TNFR1 interaction. As shown in Figure 3d, Grb2 binds to TNFR1 in the presence or absence of TNF. TNF-mediated Grb2-TNFR1 interaction is strengthened in the presence of Y401D substitution.

### 3.3. TNFR1 Y360 phosphorylation positively impacts Akt activation while Y401 phosphorylation appears to have an opposite role

**Figure 4:**
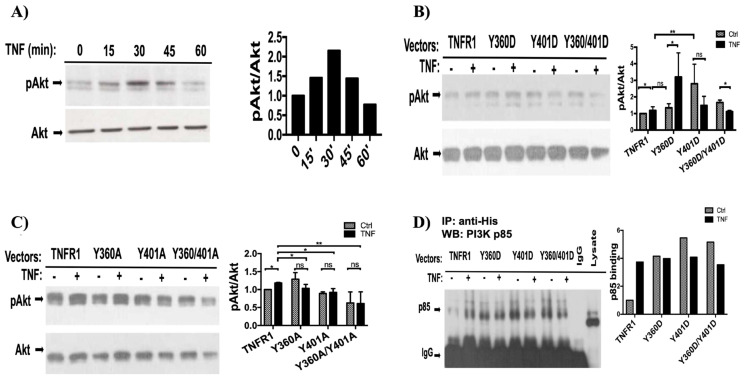
Tyrosine phosphorylation of TNFR1 positively contributes to TNF-α-induced AKT activation. a) Pattern of TNF-induced Akt activation was explored on 293T cell lysates obtained after TNF-α treatment for indicated time points. Thirty min TNF treatment resulted in maximum Akt activation. b) pAkt and Akt immunoblotting was performed on lysates obtained from 293T cells transfected with aspartic acid mutants of tyrosine phosphorylation sites. c) pAkt and Akt immunoblotting was performed on lysates obtained from 293T cells transfected with Alanine mutants of tyrosine phosphorylation sites. d) Wild type and phosphomimetic mutants of TNFR1 ectopically expressed in 293T cells treated with or without 10ng/mL TNF treatment were immunoprecipitated and blots labeled with anti-p85 antibody. For a, b, and c, Akt activation was determined by pAkt band intensities divided by Akt band intensities. _*_: p < 0.05, _**_: p < 0.01, ns: p > 0.05.

In our previous studies, we have demonstrated that despite the lack of YxxM motif, TNFR1 interacts with PI3K p85 subunit and induces AKT phosphorylation (Ozes et al., 1999; Ozes et al., 2001). Although TNF-induced Akt phosphorylation can be partly explained by Ras-induced PI3K activation (Rodriguez Viciana et al., 1994; Rodriguez Viciana et al., 1996), it was unclear whether tyrosine phosphorylation of TNFR1 could play role in PI3K pathway activation. Therefore, we explored whether tyrosine phosphorylation of TNFR1 impacts TNFα-induced Akt activation. According to our time-course experiments, in 293T cells, TNF induced transient Akt phosphorylation (T308/S473) with a maximum of 2.2-fold after 30 min (Figure 4a). Thus, transfected 293T cells were first serum starved and then treated with TNFα for 30 min to examine the impact of mutants on Akt activation. Although endogenous TNFR1 was capable of inducing 2.2 fold Akt phosphorylation, TNF treatment induced only 50% induction of Akt phosphorylation in TNFR1 and Y360D expressing cells. Contrary to ERK activation, Y401D and double mutant negatively affected TNF-induced Akt phosphorylation although both mutants significantly induced background Akt phosphorylation compared to TNFR1 expressing cells. However, all Alanine mutants suppressed TNF-induced Akt phosphorylation (Figures 4b and 4c), indicating that tyrosine phosphorylation of TNFR1 contributes to activation of PI3K pathway and Y360 phosphorylation stimulates this.

Next, we sought to determine whether the differential effects of TNFR1 mutants on Akt phosphorylation was due to altered binding of PI3K p85 subunit to TNFR1 signaling complex. When we immunoprecipitated TNFR1, we observed augmented basal (i.e. without TNF treatment) p85 binding in all D mutants compared to wild type TNFR1 (Figure 4d). In Y401D mutant, TNF stimulation resulted in less PI3Kp85 binding, consistent with suppressed Akt activation in the same mutant.

### 3.4. Impact of TNFR1 tyrosine phosphorylation on cell proliferation and NF-κB activation

**Figure 5:**
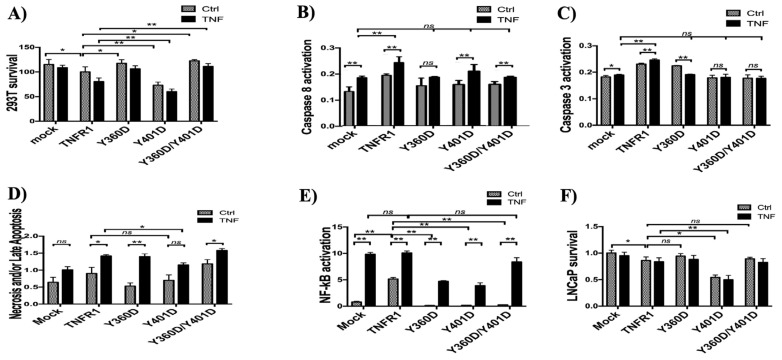
Functional consequences of TNFR1 tyrosine phosphorylation. a, f) 293T cells (a) or LNCaP cells (f) were transfected with empty pcDNA3.1a (mock), wild type TNFR1 or TNFR1 site mutants. Twenty-fourh posttransfection, cells were treated with either fresh media (n = 5) or fresh media+TNF (10ng/mL final concentration, n = 5) for 24 h. Cell survival was measured by spectrophotometric MTT assays. b,c) For measurement of apoptosis induction, 24 h posttransfection, 293T cells were treated with 10ng/mL TNF for 8 h. Caspase 8 (b) and Caspase 3 (c) activities were measured by colorimetric caspase assays. d) BrdU labeled 293T cells were transfected with TNFR1 mutants and treated with 10ng/mL TNF for 24 h. Necrosis was measured with BrdU labeled DNA ELISA on cellular supernatants. e) Mock vector, wild type TNFR1 or TNFR1 mutants were cotransfected with NF-luc reporter vector for 24 h, followed by 6 h treatment with fresh media (n = 5) or fresh media+TNF (10ng/mL final concentration, n = 5). Luciferase measurements were normalized to concurrent MTT readings to eliminate cell number-caused discrepancy. Data are represented as mean ± SEM (a–e). Statistical analysis was performed by Mann-Whitney U test. _*_: p < 0.05, _**_: p < 0.01, ns: p > 0.05.

Finally, to investigate the functional consequences of TNFR1 tyrosine phosphorylation, we compared the survival rates of wild type and mutant TNFR1 transfected cells. Transfection of wild-type TNFR1, by itself, significantly reduced the survival of 293T cells, and after 24 h treatment with 10ng/mL TNF, 293T cell survival was further reduced (Figure 5a). In Y360D and Y360D/Y401D transfected cells, on the other hand, survival of 293T cells was comparable with mock transfected cells, and significantly higher than wild type TNFR1 transfected cells. In contrast, Y401D transfection drastically decreased the survival rate (to approximately 70% of wild-type TNFR1-transfected and 50% of mock-transfected cells). Surprisingly, survival rate was further lowered by 24 h TNF treatment. In order to understand whether this decrease in cell survival in Y401D mutant was due to increased apoptosis, we sought to determine the influences of TNFR1 mutants on TNF-induced caspase-8 and caspase-3 activations (Figures 5b, 5c). Eight hour treatment with 10ng/mL TNF induced caspase 3 activation in both mock-transfected and wild-type TNFR1 transfected cells, while Y401D- and Y360D/Y401D-transfected cells were not responsive (Figure 5c). Intriguingly, TNF-induced caspase-8 activation was observed in these cells (Figure 5b). Although transfections with wild-type TNFR1 or Y360D mutant resulted in a significant increase of basal caspase-3 activation (without TNF treatment), transfections with Y401D and Y360D/Y401D mutants did not increase the basal caspase-3 activation. Interestingly, treatment of Y360D-transfected cells with TNF decreased caspase-3 activation (Figure 5c), while caspase-8 activation was not responsive to TNF (Figure 5b). In all tyrosine-to-aspartate mutants we tested, caspase-8 and caspase-3 activation following 8 h TNF treatment was similar to the TNF-induced levels of mock-transfected cells, not of wild-type TNFR1-transfected cells. Therefore, mimicking tyrosine phosphorylation of the TNFR1 death domain abrogated TNF-induced caspase activation, and in turn apoptosis. As Y401D mutant did not cause increased apoptosis, we next questioned whether the decreased survival of Y401D-transfected cells was due to increased necroptosis. Intriguingly, we observed similar necrosis rates in cells transfected with wild-type or Y401D-mutated TNFR1. Although TNFR1-transfected 293T cells were responsive to TNF treatment in inducing necrosis, Y401D mutant-transfected cells were not, resulting in decreased TNF-induced necrosis in Y401D transfected cells compared to wild-type transfected cells (Figure 5c). Therefore, the decreased survival of Y401D transfected cells was not caused by increased apoptosis or necrosis. Indeed, Y401D-transfected cells are less prone to apoptosis or necroptosis induction compared to wild-type transfected cells.

Next, since activation of NF-κB pathway can promote proliferation (Guttridge et al., 1999), we questioned whether Y401D-transfected cells had a defective NF-κB response. Expectedly, we observed more than 50% attenuation of NF-κB activity in Y401D-transfected cells compared to the ones transfected with wild-type TNFR1 (Figure 5d). However, although lowest NF-κB activity was observed in Y401D-transfected cells, the difference between Y360D and Y401D-transfected cells was not statistically significant. To examine whether the influence of Y401D mutant on cell survival was specific to 293T cells or would be observed in another cell line, we repeated MTT test in LNCaP cell line. LNCaP MTT results were parallel to what we observed in 293T cells (Figure 5e), with significantly reduced survival in LNCaP cells transfected with Y401D mutants (Figure 5e).

## 4. Discussion

TNFR1 is the ubiquitously expressed and predominantly used receptor for TNF, and is involved in many pathological and physiological events (Gough and Myles 2020). In the past 20+ years, many studies have been conducted in an effort to elucidate the molecular mechanism of TNF signaling, and these studies have taken death-domain as a starting point to show sequential bindings of signaling components to TNFR1 or one another (Gough and Myles 2020). The conclusion drawn from all these studies was the formation of a complex in the order of TNF-TNFR1-TRADD-TRAF2-cIAP1/2-RIP1/3-NEMO-TAK1-IKKs. As a consequence, activated IKK induced NF-kB and TAK1-mediated phosphorylation events of MKK4/7 and MKK3/6 resulted in activation of JNKs and p38 kinases. Although regulation of TNFR1 signaling by its binding partners’ posttranslational modifications has been extensively studied, information on how TNFR1 phosphorylation events could alter its signaling is limited. Guan et al. (Guan et al., 2011) have shown that SXXE/D motif of TNFR1 is phosphorylated and this was required for the formation of TNFR1-TRADD complex and subsequent activation of NF-kB. In addition, in 2000 Van Linden et al. (Van Linden et al., 2000) showed that TNFR1 was transiently phosphorylated by ERK at T236 and S270, and phosphorylation at these sites in turn enabled subsequent phosphorylation of TNFR1 at S240 and S244, however the authors did not study functional significance of these phosphorylation events. Following this report, in 2004, Gambelli et al. (Gambelli et al., 2004) confirmed ERK-mediated phosphorylation of TNFR1, without identifying or mentioning the actual sites, and they showed ERK-mediated phosphorylation of TNFR1 protected cells from undergoing TNF-induced apoptosis. In addition, we have previously demonstrated PKA-mediated phosphorylation of TNFR1 as a regulatory mechanism (Hapil et al., 2020).

When we inspect the juxta-membrane region of TNFR1, Figure 1a, we see that there are four S/TP motifs, next to JAK2-binding site (PLAPNP), which are phosphorylation sites for all MAPKs, while there are no SP/TP motif in the death-domain. Results of Van Linden and Gambelli can be interpreted such that ERK-mediated phosphorylation events of TNFR1 at juxta-membrane region could interfere with binding of JAK2 and/or the formation of complex-1 or apoptosis-inducing complexes. Therefore, it seems logical to suggest that ERK-mediated phosphorylation events could be used as negative-feedback loop mechanism to terminate TNF signaling and protect cells from undergoing apoptosis.

TNF-mediated activations of JAK family members (JAK1, JAK2, Tyk2) were demonstrated in 1998 (Guo et al., 1998). We have previously shown that JAK2, c-Src, and PI3Kp85 constitutively associated with TNFR1, and TNF stimulation further induced their binding (Pincheira et al., 2008). Although that study has demonstrated a strong correlative association between JAK2, c-Src, and PI3Kp85 in TNF-induced activation of p38, JNK, Akt and Stat3 pathways, it has not provided sufficient genetic and biochemical evidence for whether these bindings introduce any biochemical modifications on the structure of TNFR1. Here we demonstrate, for the first time, that TNF induces time-dependent tyrosine phosphorylation of TNFR1, and this phosphorylation can be performed by JAK2 and c-Src. Since inhibition of JAK2 augmented c-Src-mediated tyrosine phosphorylation of TNFR1, our results also demonstrated a prohibitor role of JAK2 on c-Src mediated phosphorylation of TNFR1.

Given that JAK2 and c-Src constitutively bind to TNFR1 and directly phosphorylate TNFR1, we sought to determine whether these phosphorylation events are used for transmission of signals and activation of ERK and Akt kinases. Previously, Eberhard Hildt and Stefanie Oess have shown the involvement of Grb2 in TNF-induced ERK activation (Hildt and Oess 1999). However, they concluded that Grb2 binding to C-terminal PPAP was necessary but not sufficient for full activation of ERK in response to TNF. In line with this, when we converted P_448_ and P_451_ to Alanine in Grb2 binding motif (P_448_PAP_451_) and ectopically expressed this mutant, it diminished TNF-induced ERK phosphorylation by only 35%, indicating that additional modifications on TNFR1 were needed for optimum ERK phosphorylation following TNF stimulation (Copuroglu et al., 2021). To confirm whether tyrosine phosphorylation events of Y360 and Y401 have an effect on ERK phosphorylation, we ectopically expressed phosphomimetic mutants of both sites and found that Y401D mutant increased background ERK phosphorylation by 6-fold, and TNF stimulation further increased this. However, Y360D mutant showed inhibitory effect for ERK phosphorylation, and this effect seems to have a dominant-negative role.

In addition to inducing ERK phosphorylation, Ras also directly binds to PI3Kp110 and activates PI3K pathway resulting in Akt phosphorylation (Rodriguez Viciana et al., 1994; Rodriguez Viciana et al., 1996). Therefore, we postulated that our phosphomimetic mutants could also positively stimulate TNF-induced Akt phosphorylation. Indeed, phosphomimetic mutants induced background Akt phosphorylation, whereas Alanine mutants suppressed TNF-induced Akt phosphorylation events. Since phosphorylation of TNFR1 on Y360 residue seems to positively impact Akt activation, herein reported results provide a reason for the suppression of TNF-mediated Akt activation by c-Src inhibitor treatment, which was demonstrated in a previous study (Lee et al., 2001).

Since ERK and Akt phosphorylation events are initiated by binding of Grb2 and p85 to TNFR1 respectively, we sought to determine whether our phosphomimetic mutants would create a docking site for Grb2 and PI3Kp85 on TNFR1. When we immunoprecipitated the ectopically expressed wild type and phosphomimetic mutants using anti-His antibody, we found that PI3Kp85 binding was significantly induced by TNF stimulation. More importantly, binding of PI3Kp85 to phosphomimetic mutants were stronger than its binding to wild type TNFR1 even after TNF stimulation. Ironically, TNF stimulation resulted in dissociation of PI3Kp85 from mutant receptors.

**Figure 6:**
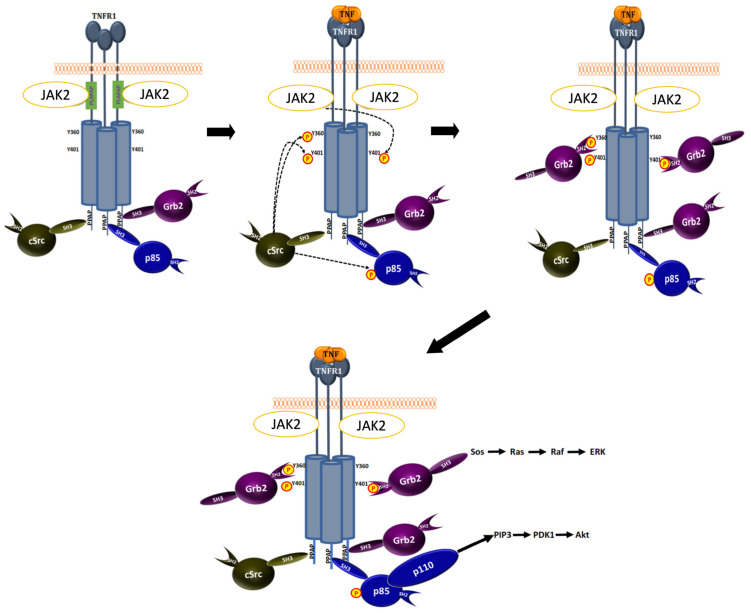
Model for “noncanonical TNF signaling”. SH3-bearing c-Src, PI3Kp85 and Grb2 bind to the SH3-recognition sequence (PPAP), JAK2 binds to juxta-membrane PLAAPAP sequence of TNFR1, constitutively. Binding of TNF to TNFR1 induces conformational change in the structure of TNFR1 such that c-Src phosphorylates Y360, Y401, and PI3Kp85 and Jak2 phosphorylates Y401 on the same or adjacent TNFR1. These phosphorylation events create binding-sites for SH2-bearing Grb2 and relieve inhibitory effect of PI3Kp85 on PI3Kp110, resulting in activation of Grb2-Sos-Ras-Raf-Erk and PI3K-PIP3-PDK1-Akt pathways.

Collectively, our results demonstrate that TNF stimulation results in tyrosine phosphorylation of TNFR1 by JAK2 and c-Src. Since TNFR1 does not bear PI3Kp85-binding motif, YXXM, and TNFR1 was shown to constitutively bind Grb2, PI3Kp85 and c-Src (Pincheira et al., 2008), we believe that this can be achieved by the interaction of SH3 domains of Grb2, PI3Kp85, c-Src with the PPAP sequence of TNFR1. After TNF stimulation, tyrosine phosphorylation of Y360 and Y401 create additional docking sites for SH2 domain of Grb2 and c-Src. As shown previously, c-Src can phosphorylate PI3Kp85 and this relieves the inhibitory activity of p85 on phosphatidylinositol-3-kinase (Cuevas et al., 2001; Daigle et al., 2002). Therefore, we believe that conformational change of TNFR1 upon TNF stimulation could enable c-Src to phosphorylate PI3Kp85, and this can induce activation of PI3K pathway resulting in augmentation of Akt phosphorylation. Similarly, induced binding of Grb2 to p-Y360 and p-401 can augment the activation of Grb2-Sos-Ras-Raf-ERK pathway, which unveils a “non-canonical TNF signaling” pathway (Figure 6).

It is of importance to note that in this report, the interaction and activation experiments were performed in a single cell line, 293T. In order to generalize the findings reported by us, validation studies in different cell lines and primary cells from model organisms are required. In this study, we provide a limited information on the influence of TNFR1 tyrosine phosphorylation events on cell proliferation, apoptosis, and NF-kappa B activation. In order to better understand the impact of these phosphorylation events on TNFR1 canonical pathway, future studies are needed to investigate the influence of TNFR1 site mutants on: i) phosphorylation by each kinase, ii) the subcellular localization of TNFR1, and iii) the physical interaction with the well-known binding partners, such as TRADD and RIP1.

## Figures and Tables

**Table 1:** Mutagenesis primers.

Name of the Primer	Sequence
Y360A-Forward	5′-GACCCCGCGACGCTGGCCGCCGTGGTGGAGAAC-3′
Y360A-Reverse	5′-GTTCTCCACCACGGCGGCCAGCGTCGCGGGGTC-3′
Y360D-Forward	5′-GACCCCGCGACGCTGGACGCCGTGGTGGAGAAC-3′
Y360D-Reverse	5′-GTTCTCCACCACGGCGTCCAGCGTCGCGGGGTC-3′
Y401A-Forward	5′-CTGCGCGAGGCGCAAGCCAGCATGCTGGCGACC-3′
Y401A-Reverse	5′-GGTCGCCAGCATGCTGGCTTGCGCCTCGCGCAG-3′
Y401D-Forward	5′-CTGCGCGAGGCGCAAGTCAGCATGCTGGCGACC-3′
Y401D-Reverse	5′-GGTCGCCAGCATGCTGACTTGCGCCTCGCGCAG-3′

**Table 2:** Mutants generated for studying the impact of tyrosine phosphorylation on TNFR1 signal transduction.

Mutant	Function
**Y360A**	Tyrosine 360 is substituted with Alanine to prevent phosphorylation, but Y401 can still be phosphorylated
**Y401A**	Tyrosine 360 can still be phosphorylated, but Y360 phosphorylation is prevented by substitution with Alanine.
**Y360A/Y401A**	Both Y360 and Y401 substituted with Alanine, preventing tyrosine phosphorylation on DD.
**Y360D**	Tyrosine 360 is substituted with Aspartic Acid (D) to mimic phosphorylation of this residue
**Y401D**	Tyrosine 401 is mutated to Aspartic Acid (D) to mimic phosphorylation of this residue
**Y360D/Y401D**	Both Y360 and Y401 are mutated to Aspartic Acid (D) to mimic double phosphorylation
